# Blood Uric Acid Prediction With Machine Learning: Model Development and Performance Comparison

**DOI:** 10.2196/18331

**Published:** 2020-10-08

**Authors:** Masuda Begum Sampa, Md Nazmul Hossain, Md Rakibul Hoque, Rafiqul Islam, Fumihiko Yokota, Mariko Nishikitani, Ashir Ahmed

**Affiliations:** 1 Department of Advanced Information Technology Kyushu University Fukuoka Japan; 2 Department of Marketing Faculty of Business Studies University of Dhaka Dhaka Bangladesh; 3 School of Business Emporia State University Kansas, KS United States; 4 Medical Information Center Kyushu University Hospital Fukuoka Japan; 5 Institute of Decision Science for a Sustainable Society Kyushu University Fukuoka Japan

**Keywords:** blood uric acid, urban corporate population, machine learning, noncommunicable diseases, Bangladesh, boosted decision tree regression model

## Abstract

**Background:**

Uric acid is associated with noncommunicable diseases such as cardiovascular diseases, chronic kidney disease, coronary artery disease, stroke, diabetes, metabolic syndrome, vascular dementia, and hypertension. Therefore, uric acid is considered to be a risk factor for the development of noncommunicable diseases. Most studies on uric acid have been performed in developed countries, and the application of machine-learning approaches in uric acid prediction in developing countries is rare. Different machine-learning algorithms will work differently on different types of data in various diseases; therefore, a different investigation is needed for different types of data to identify the most accurate algorithms. Specifically, no study has yet focused on the urban corporate population in Bangladesh, despite the high risk of developing noncommunicable diseases for this population.

**Objective:**

The aim of this study was to develop a model for predicting blood uric acid values based on basic health checkup test results, dietary information, and sociodemographic characteristics using machine-learning algorithms. The prediction of health checkup test measurements can be very helpful to reduce health management costs.

**Methods:**

Various machine-learning approaches were used in this study because clinical input data are not completely independent and exhibit complex interactions. Conventional statistical models have limitations to consider these complex interactions, whereas machine learning can consider all possible interactions among input data. We used boosted decision tree regression, decision forest regression, Bayesian linear regression, and linear regression to predict personalized blood uric acid based on basic health checkup test results, dietary information, and sociodemographic characteristics. We evaluated the performance of these five widely used machine-learning models using data collected from 271 employees in the Grameen Bank complex of Dhaka, Bangladesh.

**Results:**

The mean uric acid level was 6.63 mg/dL, indicating a borderline result for the majority of the sample (normal range <7.0 mg/dL). Therefore, these individuals should be monitoring their uric acid regularly. The boosted decision tree regression model showed the best performance among the models tested based on the root mean squared error of 0.03, which is also better than that of any previously reported model.

**Conclusions:**

A uric acid prediction model was developed based on personal characteristics, dietary information, and some basic health checkup measurements. This model will be useful for improving awareness among high-risk individuals and populations, which can help to save medical costs. A future study could include additional features (eg, work stress, daily physical activity, alcohol intake, eating red meat) in improving prediction.

## Introduction

### Background

Noncommunicable diseases such as cancer, diabetes, stroke, and cardiovascular diseases are the leading cause of death, disability, and morbidity worldwide. Surprisingly, the burden is particularly high in developing countries, accounting for 80% of deaths. In developing countries, 29% of noncommunicable disease–related deaths occur in the working-age population (aged <60 years) [1]. Therefore, noncommunicable diseases have become a major concern for developing countries and are also recognized as a threat for younger people [2]. Thus, reducing the incidence of noncommunicable diseases is one of the targets of sustainable development goals [3].

Uric acid is associated with several noncommunicable diseases such as cardiovascular disease and its risk factors, including chronic kidney disease, coronary artery disease, stroke, diabetes, metabolic syndrome, vascular dementia, and hypertension [4,5]. Uric acid is considered to be one of the predictors of various chronic diseases [6]. Hypertension showed positive correlations with uric acid levels among arsenic-endemic individuals in Bangladesh [7]. Another study found significant associations between uric acid and BMI, overweight, and waist circumference among the adult population of Bangladesh [8].

People working in urban areas, especially in private sectors, have significant workloads and remain seated for a long time to complete their tasks, and are thus more likely to develop noncommunicable diseases. In addition, there are few opportunities to engage in physical activities for the urban population of Bangladesh because of a lack of playgrounds, parks, walkable footpaths, and safe roads for cycling [9]. The prevalence of risk factors for developing noncommunicable diseases is also higher among urban than rural people in Bangladesh [9]. Therefore, it is important to control and prevent the severity of noncommunicable diseases by getting regular health checkups. However, most people are not interested in spending money and time on preventive health care services. Corporate people in Bangladesh lack health insurance and high health awareness, do not get routine mandatory health checkups, and are not habituated to use information and communications technology (ICT)-based health care services. Moreover, to get a checkup, they need to visit a hospital in traffic-congested areas and wait in a long, laborious queue [10].

The health status of an individual strongly depends on uric acid, which is considered to be a risk factor for the development of noncommunicable diseases [6,11]. Therefore, uric acid should be measured routinely at basic health checkups. As the reduction of noncommunicable diseases management cost is the main goal of health policies [12], studies are needed to determine blood uric acid regularly in a cost-effective manner. An accurate predictive model can help to identify a high-risk population without having to directly measure uric acid [13]. Using a prediction model designed by machine-learning approaches to test individual uric acid measurement rapidly will save costs and time of both doctors and patients.

However, to our knowledge, the application of machine-learning approaches for uric acid prediction in developing countries is very rare. In addition, different algorithms will work differently on different types of data with respect to various diseases such as different types of cancers and diabetes; therefore, separate investigations are needed for different types of data to identify the most accurate algorithms [14].

Machine-learning methods have not been practically established for clinical data from developing countries such as Bangladesh. There is also a lack of research on predicting blood uric acid based on basic clinical tests, dietary information, and sociodemographic characteristics using machine-learning approaches in Bangladesh, especially for the urban corporate population.

Therefore, the aim of the present study was to use machine-learning approaches to predict blood uric acid based on basic health checkup test results, dietary information, and sociodemographic characteristics. We tested several machine-learning approaches to evaluate the predictive power of these techniques and to best predict personalized uric acid measurement. Predicting health checkup test measurements is expected to be helpful in reducing health management costs.

### Existing Related Studies

During the past few decades, the prevalence of hyperuricemia has been increasing rapidly all over the world [8]. Similar to the case of developed countries, hyperuricemia is also prevalent in developing countries [15,16]. A purine-enriched diet, obesity, and alcohol intake have been reported as the main predictors of hyperuricemia [17-19]. Approximately two-thirds of the uric acid is derived from the metabolism of endogenous purine, and the remainder is a result of eating purine-enriched foods [8,20,21]. Many previous studies identified relationships between uric acid and hypertension. For example, increasing levels of serum uric acid were associated with hypertension [4]. Serum uric acid was positively associated with incident hypertension [22] and the development of hypertension [23].

Several techniques have been proposed for the survivability analysis of various cancers [24]; however, the results of machine-learning algorithms may change due to different databases and for different measuring tools [25]. One study predicted lung cancer survival time using supervised machine-learning regression predictive techniques; although the root mean squared error (RMSE) value for each model was large (>15.30), it was unclear which predictive model would yield more predictive information for lung cancer survival time [26]. Another study also predicted hyperuricemia based on basic health checkup tests in Korea using machine-learning classification algorithms, which showed poor accuracy [6]. Targeting the prediction as a continuous target, rather than a classification into categories or levels, could help to improve such predictions. Further, to make the prediction more accurate, it is necessary to incorporate more new features than traditionally used [27].

Most of the previous studies on uric acid have been conducted in selected White populations of North America and Europe or in entirely Black populations from South Africa [15]. Moreover, most of the previous machine learning–based research in health care has been conducted in developed countries [28]. However, there has been minimal application of supervised machine learning for medical data to predict diseases, survivability of diseases, and different types of health checkup test results using sample data from developing countries such as Bangladesh.

### Study Objectives and Design

We used machine-learning approaches for development of a predictive model because clinical input data are not completely independent and complex interactions exist between them. Conventional statistical models have limitations to consider these complex interactions, whereas machine learning can consider all possible interactions among input data. Machine-learning prediction models can incorporate all of the input variables with marginal effect and variables with unknown associations with the targeted outcome variable. Machine-learning algorithms are used to identify patterns in datasets and to iteratively improve the performance of this identification with additional data [26]. Machine-learning algorithms have been extensively used in various domains such as in advertisement, agriculture, banking, online shopping, insurance, finance, social media, travel, tourism, marketing, consumer behavior, and fraud detection. These approaches are also used to analyze current and historical facts to make predictions about future events. Machine learning has also been used in the health care field for the prevention, diagnosis, and treatment phases of various diseases such as diabetes, cancer, cardiology, and mental health [29,30]. Through machine-learning prediction models, we incorporated both well-known risk factors of high uric acid such as age, BMI, and blood glucose, along with factors without clear associations to uric acid [6].

## Methods

### Sample

Data were collected from employees who work in the Grameen bank complex of Dhaka, Bangladesh. The Grameen bank complex comprises 18 different institutions such as Grameen Bank, Grameen Communications, other nongovernment organizations, and private companies, with more than 500 workers. We collected data from 271 employees who received human-assisted Portable Health Clinic (PHC) system services to predict blood uric acid. In general, a large sample size is required for machine-learning approaches. However, some studies have used a small sample size, including N=300 [27] and N=118 [31]. Of note, a small sample size has also been associated with higher classification accuracy [32].

Grameen Communications, Bangladesh and Kyushu University, Japan have jointly developed a human-assisted PHC system [33]. A PHC is an eHealth system that aims to provide affordable primary health care services to prevent the severity of or to control noncommunicable diseases. A PHC system has four modules: (1) a set of medical devices, (2) a software system to collect and archive medical records, (3) health care workers to make the clinical measurements and explain ePrescriptions, and (4) ICT-trained call center doctors. Consumers come to the service point and a health checkup is conducted by pretrained health care workers. If needed, the consumer is connected to the call center doctors for a consultation. The clinical measurements addressed by a PHC are as follows: (1) blood pressure; (2) pulse rate; (3) body temperature; (4) oxygenation of blood (SpO_2_); (5) arrhythmia; (6) BMI; (7) waist, hip, and waist/hip ratio; (8) blood glucose; (9) blood cholesterol; (10) blood hemoglobin; (11) blood uric acid; (12) blood grouping; (13) urinary sugar; and (14) urinary protein.

These test items (except arrhythmia, blood cholesterol, blood hemoglobin, blood grouping, urinary sugar, and urinary protein because there were many missing cases in these measurements) in this PHC system were used as input factors for the present study, and uric acid measurement was set as an output factor.

### Measurements

Clinical measurements were obtained through direct diagnosis using PHC instruments operated by well-trained nurses or health care professionals. Data on dietary information and sociodemographic characteristics were collected during interviews using a standard questionnaire.

### Regression Predictive Modeling

As the targeted output variable of this study is a continuous variable, the regression predictive model was applied, and our objective was to predict the value of the blood uric acid of an individual. Among the multiple types of regression predictive models available, it is important to choose the best-suited models based on the type of independent and dependent variables, dimensionality in the data, and other essential characteristics of the data. We selected several algorithms that showed the best performance. Overall, no specific algorithm works best for every problem, which is especially true in the case of machine learning (ie, predictive modeling). For example, it cannot be stated that neural networks are always better than decision trees or vice versa. There are many factors at play, such as the size and structure of the dataset. Therefore, in this study, we used several machine-learning approaches, including boosted decision tree regression, decision forest regression, neural network, Bayesian linear regression, and linear regression, to predict personalized blood uric acid values based on basic health checkup test results, dietary information, and sociodemographic characteristics. We chose these five specific machine-learning algorithms because they are popular tools used to predict clinical data and they are widely used regression predictive models. These five models are also traditional machine-learning models, which perform well for regression tasks [26], and have been applied in other studies on biomedical data prediction [34].

Because a regression predictive model predicts a quantity, the performance of the model must be reported as an error in the predictions. Among the many evaluation criteria to estimate the performance of a regression predictive model, the most common approach is to calculate the RMSE.

These five models were chosen for comparison in this study owing to their popularity in medical data prediction. Therefore, we compared these algorithms to see if the prediction accuracy can be further improved. Details of each model are described below.

#### Boosted Decision Tree Regression

Gradient boosting methods are a family of powerful machine-learning methods that have shown considerable success in a wide range of practical applications [35]. This model is particularly well suited for making predictions based on clinical data and exhibits high performance on clinical data [13,26,36,37]. Boosting is a popular machine-learning ensemble method [38]. Boosting means that each tree is dependent on prior trees. The algorithm learns by fitting the residual of the trees that preceded it; thus, boosting in a decision tree ensemble tends to improve accuracy with some small risk of less coverage. In the Azure Machine Learning platform, boosted decision trees use an efficient implementation of the MART gradient boosting algorithm. Gradient boosting is a machine-learning technique for regression problems. It builds each regression tree in a stepwise fashion, using a predefined loss function to measure the error in each step and correct for it in the next step. Thus, the prediction model is an ensemble of weaker prediction models. In regression problems, boosting builds a series of trees in a stepwise fashion, and then selects the optimal tree using an arbitrary differentiable loss function [39]. Similar to random forest, boosting uses many smaller, weaker models and brings them together into a final summed prediction. However, the idea of boosting is to add new models to the ensemble in a sequence for several sequences. In each iteration, a new weak model is trained with respect to the whole ensemble learned up to that new model. These new models, iteratively produced, are built to maximally correlate with the negative gradient of the loss function that is also associated with the ensemble as a whole. In this approach, a performance function is placed on the gradient boosting machine to find the point at which adding more iterations becomes negligible in benefit (ie, when adding more simple models, decision trees no longer reduce the error by a significant margin). It is at this point that the ensemble sums all of the predictions into a final overall prediction [26].

#### Decision Forest Regression

Decision forest or random forest has been employed in many biomedicine research applications [40-42]. In the regression problem, the decision forest output is the average value of the output of all decision trees [42-44]. Decision forests compare favorably to other techniques [45]. This regression model consists of an ensemble of decision trees. A collection of trees constitutes a forest. Each tree in a regression decision forest outputs a Gaussian distribution as a prediction. Aggregation is performed over the ensemble of trees to find a Gaussian distribution closest to the combined distribution for all trees in the model [45]. This technique generates several decision trees during training, which are allowed to split randomly from a seed point. This results in a “forest” of randomly generated decision trees whose outcomes are ensembled by the random forest algorithm to achieve more accurate prediction than possible with a single tree. One problem with a single decision tree is overfitting, making the predictions seem very good on the training data, but unreliable in future predictions [26]. By using decision forest regression, we can train a model with a relatively small number of samples and obtain good results.

#### Neural Network

Applying a neural network to the problem can provide much more prediction power compared to a traditional regression. Neural networks have the highest accuracy in predicting various health conditions such as heart attack and heart diseases [46,47], and have become widely used machine-learning algorithms. The neural network is a network of connected neurons. The neurons cannot operate without other neurons to which they are connected. Usually, these neurons are grouped in layers and process data in each layer, which are then passed forward to the next layers. The last layer of neurons makes decisions. The basic neural network, which is also known as multilayer perceptron, is used for comparison with one hidden layer of 500 neurons that is considered to be a reasonable number in neural network–based approaches [48].

#### Bayesian Linear Regression

Bayesian linear regression is the Bayesian approach to linear regression analysis. Bayesian regression methods are very powerful, as they not only provide point estimates of regression parameters but also deliver an entire distribution over these parameters. In recent years, Bayesian learning has been widely adopted and was even proven to be more powerful than other machine-learning techniques [49]. Bayesian linear regression follows a fairly natural mechanism to survive insufficient data or poorly distributed data by placing a prior on the coefficients and on the noise so that the priors can take over in the absence of data. Bayesian linear regression provides information about which parts of the model fit confidently to the data and which parts are very uncertain. The result of Bayesian linear regression is a distribution of possible model parameters based on the data and the prior. This enables quantifying the uncertainty about the model; if there are fewer data points, the posterior distribution will be more spread out.

#### Linear Regression

Linear regression is one of the most well-known and well-understood algorithms in statistics and machine learning. It is a fast yet simple algorithm to test, which is suitable for continuous dependent variables and can be fitted with a linear function (straight line). Linear regression models have been widely applied to predict medical data [50]. Linear regression is a very simple machine-learning method in which each data point consists of a pair of vectors: the input vector and the output vector. As the simplest, oldest, and most commonly used correlational method, linear regression fits a straight line to a set of data points using a series of coefficients multiplied to each input (ie, a weighting function) and an intercept. The weights are decided within the linear regression function in such a way that minimizes the mean error. These weight coefficients multiplied by the respective inputs, plus an intercept, give a general function for the outcome (in this case, uric acid measurement). Thus, linear regression is easy to understand and quick to implement, even on larger datasets. The disadvantage of this method is that it is inherently linear and does not always fit real-world data [26].

### Model Performance Comparison

In this study, we used five machine-learning algorithms that have been used in previous studies to predict several health conditions, including lung cancer, diabetes, heart attack, heart diseases, and breast cancer. Therefore, we considered the above five regression algorithms to be best suited for our study.

We used the Azure machine-learning platform, which is a cloud-based computing platform that allows for building, testing, and deploying predictive analytics solutions [51], to estimate the five machine-learning algorithms that are widely used to predict medical data.

For evaluating the performance of the models, RMSE values from each model were used. The RMSE of a model is the average distance between the model’s prediction and the actual outcome [26], and is considered to be the prime evaluation criterion for examining the prediction performance of a continuous dependent variable through the regression predictive technique using machine-learning algorithms [34,52]. Therefore, as we are predicting the continuous value of blood uric acid, we used the regression predictive technique and evaluated the performance of models by using the RMSE. Like classification, the regression task is inductive, with the main difference being the continuous nature of the output [45].

Many studies have used two validation methods to evaluate the capability of a model: the holdout method and k-fold cross-validation. According to the goal of each problem and the size of the data, different methods can be chosen to solve the problem. In the holdout method, as a popular validation method, the dataset is divided into two distinct parts: a training set and test set. The training set is used to train the machine-learning algorithm and the test set is used to evaluate the model [42,53]. The holdout method involves portioning the datasets into nonoverlapping subsets, where the first subset is entirely used for training and the rest for testing [54], and is often used instead of k-fold cross-validation [55-57]. When given no testing sample independent of the training sample, one can randomly select and hold out a portion of the training sample for testing, and construct a prediction with only the remaining sample. Typically, 30% of the training sample is set aside for testing and 70% is used for the training step [58-60].

In this study, the holdout method was used to evaluate the proposed model because it is more suitable for small sample sizes [61,62]. It is used in most of the machine-learning platforms, including the Azure machine learning studio [51] that was applied in our study. A random train-test split method is the recommended dataset split method, and machine-learning models in general yield more accurate results when trained with a greater amount of data points (70%:30%) [63]. Many previous studies also applied a 70%:30% random train-test split method in similar fields [63-65].

It is common practice to split the data into 70% as a training set and 30% as a testing set. This splitting ratio is large enough to yield statistically meaningful results. Train-test split is a simple and reliable validation approach. A portion of the data is split before any model development steps and is used only once to validate the developed model [32]. Therefore, in this study, each model was trained on a 70% training sample to ensure that each model was trained uniformly. We split the data according to a training set ratio of 0.7 and test set ratio of 0.3. We did not use the cross-validation method because k-fold cross-validation produces strongly biased performance estimates with small sample sizes [32].

**Figure 1:**
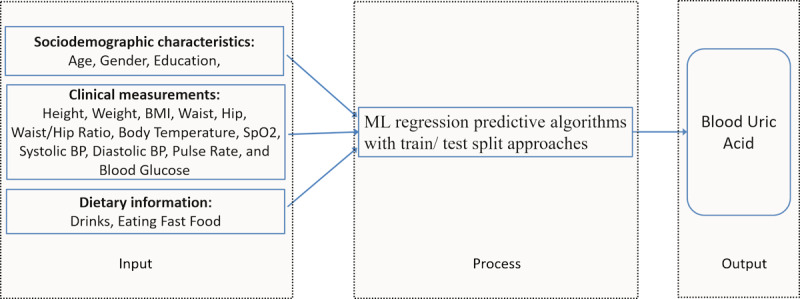
The input-process-output model used for predicting uric acid after processing 17 input variables by machine-learning (ML) algorithms. BP: blood pressure.

The input-process-output model for predicting blood uric acid based on sociodemographic characteristics, dietary information, and some basic health checkup test results is shown in Figure 1.

### Ethical Approval

We obtained ethical approval from the National Research Ethics Committee of the Bangladesh Medical Research Council (approval no. 18325022019).

## Results

### Characteristics of the Study Population

Data from a total of 271 employees of Grameen bank complex were collected during health checkups provided by the PHC service. The descriptive statistics of baseline characteristics of the participants are shown in Table 1.

The mean age of participants was 49.61 years. Most of the respondents had a BMI that put them in the category of overweight according to the World Health Organization criteria (range 25-29.9). The uric acid of the participants was borderline with a mean of 6.63 mg/dL, as the normal reference level is <7.0 mg/dL [11]. Therefore, the majority of the participants should be checking their uric acid regularly.

**Table 1:** Summary statistics of the selected continuous predictors (N=271).

Variables	Range	Mean (SD)
Age (years)	34-77	49.61 (7.39)
Height (cm)	140-184	163.05 (7.45)
Weight (kg)	44.20-114.40	67.52 (10.06)
BMI (kg/m^2^)	18.39-40.53	25.37 (3.20)
Waist (cm)	63.60-118.00	90.24 (7.80)
Hip (cm)	80.00-127.00	94.54 (6.29)
Waist/hip ratio	0.64-1.11	0.96 (0.06)
Body temperature (°F)	92.12-99.64	96.07 (1.15)
Blood oxygenation (SpO_2_) (%)	93-99	97.67 (1.17)
Systolic blood pressure (mmHg)	92-180	126.68 (14.88)
Diastolic blood pressure (mmHg)	59-108	81.71 (8.43)
Pulse rate (bpm)	51-123	80.27 (11.66)
Blood uric acid (mg/dL)	3.10-11.00	6.63 (1.54)
Blood glucose (mg/dL)	66.60-392.40	128.02 (56.92)

The lifestyle characteristics of the participants are summarized in Table 2. The majority of the respondents were male and had completed a college/university degree. Approximately 10% reported that they drink sugar-containing drinks 3 or more times a week and nearly 20% reported that they regularly eat fast food.

**Table 2:** Summary statistics of selected categorical predictors related to lifestyle factors (N=271).

Variable		n (%)
**Gender**		
	Male	225 (83.0)
	Female	46 (17.0)
**Education**		
	No education	10 (3.7)
	Primary school completed	30 (11.1)
	Secondary school completed	11 (4.1)
	High school completed	23 (8.5)
	Vocation school completed	1 (0.4)
	College/university completed	63 (23.2)
	Higher education (master or doctorate degree) completed	133 (49.1)
**Consumption of high-sugar drinks (eg, soda, fruit juice) ≥3 times a week**		
	Yes	26 (9.6)
	No	245 (90.4)
**Consumption of fast food such as pizza, hamburger, deep-fried foods (eg, singara, samosa, moglai parata) ≥3 times a week**		
	Yes	49 (18.1)
	No	222 (81.9)

### Prediction Performance

The RMSE was used to examine the prediction performance of the regression predictive technique with machine-learning algorithms. As shown in Table 3, the boosted decision tree regression model showed the best performance among the tested models.

**Table 3:** Comparison of modeling techniques ranked from best to worst based on root mean squared error (RMSE).

Model	RMSE^a^	Mean absolute error^b^	Coefficient of determination (R^2^)
Boosted decision tree regression	0.03	0.01	0.99
Decision forest regression	0.75	0.53	0.75
Neural network	1.46	1.13	0.04
Bayesian linear regression	1.37	1.06	0.16
Linear regression	1.36	1.06	0.17

^a^Root mean squared error measures the average magnitude of the error by taking the square root of the average of squared differences between predicted and actual observations. That is, it measures how close the predicted value is to the actual vale. There is no cutoff or benchmark value; the smaller the value, the better the prediction.

^b^The mean absolute error is the sum of the absolute differences between predicted and actual values.

### Score Model

**Figure 2:**
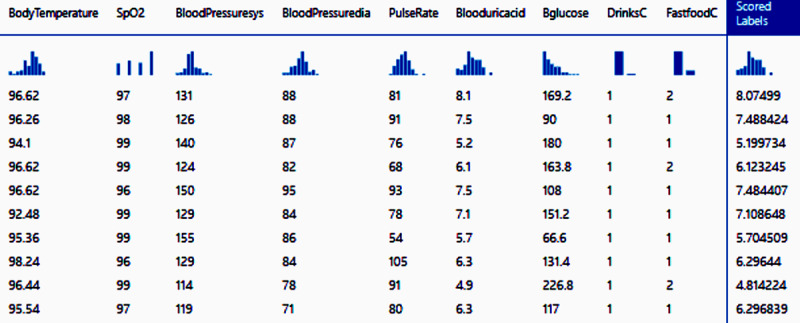
Partial view of the score model obtained by the boosted decision tree regression. Scored labels is the result column in this scoring result. The numbers are the predicted blood uric acid value for each individual.

The Score model represents the predicted value of the output or predicting variable. For regression models, the score model generates a predicted numeric value. The score model obtained using the boosted decision tree regression model is shown in Figure 2.

## Discussion

### Principal Findings

Machine-learning algorithms can identify the pattern in a dataset that may not be apparent directly. Thus, machine learning can provide useful information and support to medical staff by identifying patterns that may not be readily apparent [25]. There are several advantages of choosing machine-learning algorithms over conventional statistical methods for designing a prediction model. First, machine-learning algorithms can handle noisy information. Second, they can model complex, nonlinear relationships between variables without prior knowledge of a model [66], which enables including all information from the dataset during the analysis [6]. Finally, machine learning can consider all potential interactions between input variables, whereas conventional statistical analysis assumes that the input variables are independent [67]. Since many input variables are interrelated in complex ways, whether known or not, machine-learning algorithms can be used to identify high-risk individual cases and can help medical staff with clinical assessment [67].

Machine learning uses techniques that enable machines to use experience to improve at tasks. Through machine learning, data fed into an algorithm or model are used to train and test a model. The model is then deployed to conduct an automated rapid predictive task or to receive the predictions returned by the model. In many clinical studies, the gradient boosting machine-learning algorithm has been successfully used to predict cardiovascular diseases [13]. The gradient boosting decision tree method introduced by Friedman [68] predicted BMI with an accuracy of 0.91 [37]. In the current study, the boosted decision tree regression was found to be the best predictive model for uric acid, followed by decision forest regression. These are both popular ensemble learning methods.

In this study, a prediction model was designed for improving uric acid prediction by including not only well-known relevant factors of high uric acid such as age, gender, and BMI but also factors that have unknown associations with uric acid. The test items used in the PHC service were used as input factors, except for uric acid as the output factor. Therefore, a tool to predict uric acid was developed with good predictive performance based on the RMSE of 0.03; this RMSE is better than any previously reported in the literature in models related to biomedical data [26,35,69]. These results can provide useful insights for understanding the observed trend in population health and to inform future strategic decision making for improved health outcomes.

It is very important to compare the results of this study to previous related work. Most of the previous studies reported performance measurements as a function of classification accuracy, which may not be directly compared to this study with a regression approach to building a predictive model for a continuous variable (blood uric acid value).

A previous uric acid prediction study [6] that predicted uric acid levels based on health checkup data archived in a hospital in Korea used data that were collected from laboratory-quality devices in a very specific group of people who participated in an expensive, self-paid comprehensive health checkup program. The data were collected from 38,001 people, and the prediction sensitivity was 0.73 and 0.66 using naive Bayes classification and random forest classification models, respectively. They used a total of 25 variables available in their database. Our uric acid prediction model was developed using machine-learning approaches and included personal characteristics, dietary information, and basic clinical measurements. These data were collected using portable and cheap devices. Health records of 271 employees (aged 34-77 years with 83% men) were collected. We found that uric acid value can be predicted with an RMSE value of 0.03. Among the five machine-learning algorithms, boosted decision tree regression was found to be the most effective.

### Contribution

This is the first study aimed at predicting laboratory test results of health measurements or health checkup items in Bangladesh. The ability to determine uric acid using the developed machine-learning prediction model would avoid the need for health care workers of PHC services to carry out uric acid measurements. These findings can be helpful in achieving sustainable development goals and universal health coverage, and thus reducing overall morbidity and mortality. Using the prediction model designed by the machine-learning approaches to measure individual blood uric acid will save the cost and time of doctors as well as patients. This prediction model can also be applied to other institutions.

By inputting only 17 variables (12 basic clinical measurements, 3 sociodemographic characteristics, and 2 dietary characteristics) in the models, we were able to predict blood uric acid. In emergency situations such as floods, pandemics, tsunamis, and other contexts in which it is difficult to physically go to the clinic, blood uric acid can be predicted, therefore contributing to public health improvement. From the perspective of underdeveloped or developing countries such as Bangladesh, people do not check their blood uric acid frequently and do not know about the potential associated complications. However, people frequently measure the clinical variables that are included in the predictive models. By applying these machine-learning algorithms, we can also predict other health parameters such as blood glucose and SpO_2_. Moreover, beyond the fields of health care and medical science, similar models can also be applied to agriculture, insurance and banking, online shopping, travel and tourism, marketing, and consumer behavior along with many other fields.

### Conclusion and Prospects

This study provides a measure for reducing noncommunicable diseases, and hence can be a good component of national or global health plans. We developed a uric acid prediction model based on personal characteristics, dietary information, and some basic clinical measurements related to noncommunicable disease risk. Such a uric acid prediction model will be useful for improving awareness among high-risk individuals. The blood uric acid prediction model can further help to provide health services with the early detection and cost-effective management of noncommunicable diseases.

There are a few limitations of this study. First, the sample size was relatively small, which should be increased for training the prediction model in the future. Second, this study was limited to a particular area among a group of employees who work in a corporate setting. Our prediction model was not confirmed with data from other institutes. Although the framework achieved high performance on Grameen bank complex data, we believe that this model will also be suitable for predicting blood uric acid values in individuals that work in other types of corporate settings. Third, the included variables in the model were selected based on validated key features from previous studies rather than by using statistical approaches to identify the significant influence of factors on the output variable from the data. A future study could also include additional features (eg, work stress, everyday physical activity, eating red meat). Fourth, this study evaluated only five machine-learning algorithms among many other algorithms available. Finally, we applied only a random split method (train/test split method), although cross-validation is a good method for training and testing a dataset. We did not consider applying the cross-validation method in this case owing to the small dataset. Therefore, further study can be considered with an extended sample size and cross-validation method.

Despite these limitations, we conclude that this study represents a successful case to open discussions on further applications of this combined approach to wider regions and various types of health checkup measurements.

## Abbreviations

ICT: information and communications technology
PHC: Portable Health Clinic
RMSE: root mean squared error
SpO_2_: blood oxygen level

## References

[1] Nohara Y, Kai E, Ghosh PP, Islam R, Ahmed A, Kuroda M, Inoue S, Hiramatsu T, Kimura M, Shimizu S, Kobayashi K, Baba Y, Kashima H, Tsuda K, Sugiyama M, Blondel M, Ueda N, Kitsuregawa M, Nakashima N (2015). Health checkup and telemedical intervention program for preventive medicine in developing countries: verification study. J Med Internet Res.

[2] Khalequzzaman M, Chiang C, Choudhury SR, Yatsuya H, Al-Mamun MA, Al-Shoaibi AAA, Hirakawa Y, Hoque BA, Islam SS, Matsuyama A, Iso H, Aoyama A (2017). Prevalence of non-communicable disease risk factors among poor shantytown residents in Dhaka, Bangladesh: a community-based cross-sectional survey. BMJ Open.

[3] (2019). Goal 3: ensure healthy lives and promote well-being for all at all ages. United Nations: Sustainable Development Goals.

[4] Loeffler LF, Navas-Acien A, Brady TM, Miller ER, Fadrowski JJ (2012). Uric acid level and elevated blood pressure in US adolescents: National Health and Nutrition Examination Survey, 1999-2006. Hypertension.

[5] Feig DI, Kang D, Johnson RJ (2008). Uric acid and cardiovascular risk. N Engl J Med.

[6] Lee S, Choe E, Park B (2019). Exploration of Machine Learning for Hyperuricemia Prediction Models Based on Basic Health Checkup Tests. J Clin Med.

[7] Huda N, Hossain S, Rahman M, Karim MR, Islam K, Mamun AA, Hossain MI, Mohanto NC, Alam S, Aktar S, Arefin A, Ali N, Salam KA, Aziz A, Saud ZA, Miyataka H, Himeno S, Hossain K (2014). Elevated levels of plasma uric acid and its relation to hypertension in arsenic-endemic human individuals in Bangladesh. Toxicol Appl Pharmacol.

[8] Ali N, Perveen R, Rahman S, Mahmood S, Rahman S, Islam S, Haque T, Sumon AH, Kathak RR, Molla NH, Islam F, Mohanto NC, Nurunnabi SM, Ahmed S, Rahman M (2018). Prevalence of hyperuricemia and the relationship between serum uric acid and obesity: A study on Bangladeshi adults. PLoS One.

[9] Zaman M, Rahman MM, Rahman MR, Bhuiyan M, Karim MN, Chowdhury MA (2016). Prevalence of risk factors for non-communicable diseases in Bangladesh: Results from STEPS survey 2010. Indian J Public Health.

[10] Sampa MB, Hossain MN, Hoque MR, Islam R, Yokota F, Nishikitani M, Fukuda A, Ahmed A (2020). Influence of Factors on the Adoption and Use of ICT-Based eHealth Technology by Urban Corporate People. JSSM.

[11] Kim S, Chang Y, Yun KE, Jung H, Lee S, Shin H, Ryu S (2017). Development of Nephrolithiasis in Asymptomatic Hyperuricemia: A Cohort Study. Am J Kidney Dis.

[12] Hunter DJ, Reddy KS (2013). Noncommunicable diseases. N Engl J Med.

[13] Zhang Z, Zhao Y, Canes A, Steinberg D, Lyashevska O, written on behalf of AME Big-Data Clinical Trial Collaborative Group (2019). Predictive analytics with gradient boosting in clinical medicine. Ann Transl Med.

[14] Noohi NA, Ahmadzadeh M, Fardaei M (2013). Medical Data Mining and Predictive Model for Colon Cancer Survivability. Int J Innov Res Eng Sci.

[15] Conen D, Wietlisbach V, Bovet P, Shamlaye C, Riesen W, Paccaud F, Burnier M (2004). Prevalence of hyperuricemia and relation of serum uric acid with cardiovascular risk factors in a developing country. BMC Public Health.

[16] Chen L, Zhu W, Chen Z, Dai H, Ren J, Chen J, Chen L, Fang L (2007). Relationship between hyperuricemia and metabolic syndrome. J Zhejiang Univ Sci B.

[17] Nakanishi N, Yoshida H, Nakamura K, Suzuki K, Tatara K (2001). Predictors for development of hyperuricemia: an 8-year longitudinal study in middle-aged Japanese men. Metabolism.

[18] Wortmann RL (2002). Gout and hyperuricemia. Curr Opin Rheumatol.

[19] Ogura T, Matsuura K, Matsumoto Y, Mimura Y, Kishida M, Otsuka F, Tobe K (2004). Recent trends of hyperuricemia and obesity in Japanese male adolescents, 1991 through 2002. Metabolism.

[20] Schlesinger N (2005). Dietary factors and hyperuricaemia. Curr Pharm Des.

[21] Miao Z, Yan S, Wang J, Wang B, Li Y, Xing X, Yuan Y, Meng D, Wang L, Gu J, Zhang S, Li C, Wang C (2009). Insulin resistance acts as an independent risk factor exacerbating high-purine diet induced renal injury and knee joint gouty lesions. Inflamm Res.

[22] Mellen PB, Bleyer AJ, Erlinger TP, Evans GW, Nieto FJ, Wagenknecht LE, Wofford MR, Herrington DM (2006). Serum uric acid predicts incident hypertension in a biethnic cohort: the atherosclerosis risk in communities study. Hypertension.

[23] Perlstein TS, Gumieniak O, Williams GH, Sparrow D, Vokonas PS, Gaziano M, Weiss ST, Litonjua AA (2006). Uric acid and the development of hypertension: the normative aging study. Hypertension.

[24] Agrawal A, Misra S, Narayanan R, Polepeddi L, Choudhary A (2012). Lung Cancer Survival Prediction using Ensemble Data Mining on Seer Data. Sci Program.

[25] Delen D, Walker G, Kadam A (2005). Predicting breast cancer survivability: a comparison of three data mining methods. Artif Intell Med.

[26] Lynch CM, Abdollahi B, Fuqua JD, de Carlo AR, Bartholomai JA, Balgemann RN, van Berkel VH, Frieboes HB (2017). Prediction of lung cancer patient survival via supervised machine learning classification techniques. Int J Med Inform.

[27] Zheng T, Xie W, Xu L, He X, Zhang Y, You M, Yang G, Chen Y (2017). A machine learning-based framework to identify type 2 diabetes through electronic health records. Int J Med Inform.

[28] Gu D, Li J, Li X, Liang C (2017). Visualizing the knowledge structure and evolution of big data research in healthcare informatics. Int J Med Inform.

[29] Triantafyllidis AK, Tsanas A (2019). Applications of Machine Learning in Real-Life Digital Health Interventions: Review of the Literature. J Med Internet Res.

[30] Misawa D, Fukuyoshi J, Sengoku S (2020). Cancer Prevention Using Machine Learning, Nudge Theory and Social Impact Bond. Int J Environ Res Public Health.

[31] Zelic I, Kononenko I, Lavrac N, Vuga V (1997). Induction of decision trees and Bayesian classification applied to diagnosis of sport injuries. J Med Syst.

[32] Vabalas A, Gowen E, Poliakoff E, Casson AJ (2019). Machine learning algorithm validation with a limited sample size. PLoS One.

[33] Sampa MB, Hossain N, Hoque R, Islam R, Yokota F, Nishikitani M, Fukuda A, Ahmed A, Streitz N, Konomi S (2019). A Framework of Longitudinal Study to Understand Determinants of Actual Use of the Portable Health Clinic System. Distributed, Ambient and Pervasive Interactions. HCII 2019. Lecture Notes in Computer Science, vol 11587.

[34] Luo W, Phung D, Tran T, Gupta S, Rana S, Karmakar C, Shilton A, Yearwood J, Dimitrova N, Ho TB, Venkatesh S, Berk M (2016). Guidelines for Developing and Reporting Machine Learning Predictive Models in Biomedical Research: A Multidisciplinary View. J Med Internet Res.

[35] Natekin A, Knoll A (2013). Gradient boosting machines, a tutorial. Front Neurorobot.

[36] Afzal M, Hussain M, Malik KM, Lee S (2019). Impact of Automatic Query Generation and Quality Recognition Using Deep Learning to Curate Evidence From Biomedical Literature: Empirical Study. JMIR Med Inform.

[37] Hu M, Nohara Y, Wakata Y, Ahmed A, Nakashima N, Nakamura M (2018). Machine Learning Based Prediction of Non-communicable Diseases to Improving Intervention Program in Bangladesh. Eur J Bioinformatics.

[38] Wu J, Roy J, Stewart WF (2010). Prediction Modeling Using EHR Data. Medical Care.

[39] Manna S, Biswas S, Kundu R, Rakshit S, Gupta P, Barman S (2017). A statistical approach to predict flight delay using gradient boosted decision tree.

[40] Zhao X, Zou Q, Liu B, Liu X (2015). Exploratory Predicting Protein Folding Model with Random Forest and Hybrid Features. Curr Proteomics.

[41] Liao Z, Ju Y, Zou Q (2016). Prediction of G Protein-Coupled Receptors with SVM-Prot Features and Random Forest. Scientifica (Cairo).

[42] Zou Q, Qu K, Luo Y, Yin D, Ju Y, Tang H (2018). Predicting Diabetes Mellitus With Machine Learning Techniques. Front Genet.

[43] Liaw A, Wiener M (2002). Classification and Regression by RandomForest. R News.

[44] Svetnik V, Liaw A, Tong C, Culberson JC, Sheridan RP, Feuston BP (2003). Random forest: a classification and regression tool for compound classification and QSAR modeling. J Chem Inf Comput Sci.

[45] Criminisi A, Shotton J, Konukoglu E (2012). Decision Forests for Classification, Regression, Density Estimation, Manifold Learning and Semi-Supervised Learning. Foundations and Trends in Computer Graphics and Vision.

[46] Yahyaie M, Tarokh MJ, Mahmoodyar MA (2019). Use of Internet of Things to Provide a New Model for Remote Heart Attack Prediction. Telemed J E Health.

[47] Dangare CS, Apte SS (2012). A data mining approach for prediction of heart disease using neural networks. Int J Comput Eng Technol.

[48] Li X, Ding Q, Sun J (2018). Remaining useful life estimation in prognostics using deep convolution neural networks. Reliab Eng Syst Saf.

[49] Jihan N (2019). Bayesian Learning for Machine Learning: Linear Regression (Part 2). DZone.

[50] Zarkogianni K, Mitsis K, Litsa E, Arredondo M, Ficο G, Fioravanti A, Nikita KS (2015). Comparative assessment of glucose prediction models for patients with type 1 diabetes mellitus applying sensors for glucose and physical activity monitoring. Med Biol Eng Comput.

[51] Barga R, Fontama V, Tok WH (2015). Predictive Analytics with Microsoft Azure Machine Learning.

[52] Cox DR (1958). Two further applications of a model for binary regression. Biometrika.

[53] Kim J (2009). Estimating classification error rate: Repeated cross-validation, repeated hold-out and bootstrap. Comput Stat Data Analysis.

[54] Yadav S, Shukla S (2016). Analysis of k-Fold Cross-Validation over Hold-Out Validation on Colossal Datasets for Quality Classification.

[55] Zarkogianni K, Litsa E, Vazeou A, Nikita KS (2013). Personalized glucose-insulin metabolism model based on self-organizing maps for patients with Type 1 Diabetes Mellitus.

[56] Ruiz-Velázquez E, Alanis AY, Femat R, Quiroz G (2011). Neural modeling of the blood glucose level for type 1 diabetes mellitus patients.

[57] Mirshekarian S, Bunescu R, Marling C, Schwartz F (2017). Using LSTMs to learn physiological models of blood glucose behavior.

[58] Ben Ali J, Hamdi T, Fnaiech N, Di Costanzo V, Fnaiech F, Ginoux J (2018). Continuous blood glucose level prediction of Type 1 Diabetes based on Artificial Neural Network. Biocybern Biomed Eng.

[59] Hamdi T, Ben Ali J, Di Costanzo V, Fnaiech F, Moreau E, Ginoux J (2018). Accurate prediction of continuous blood glucose based on support vector regression and differential evolution algorithm. Biocybern Biomed Eng.

[60] Li J, Xu Q, Shah N, Mackey TK (2019). A Machine Learning Approach for the Detection and Characterization of Illicit Drug Dealers on Instagram: Model Evaluation Study. J Med Internet Res.

[61] Varoquaux G (2018). Cross-validation failure: Small sample sizes lead to large error bars. Neuroimage.

[62] Isaksson A, Wallman M, Göransson H, Gustafsson M (2008). Cross-validation and bootstrapping are unreliable in small sample classification. Pattern Recogn Lett.

[63] Livera A, Theristis M, Makrides G, Ransome S, Sutterlueti J, Georghiou GE (2019). Optimal development of location and technology independent machine learning photovoltaic performance predictive models.

[64] Polat K, Akdemir B, Güneş S (2008). Computer aided diagnosis of ECG data on the least square support vector machine. Dig Sign Process.

[65] Soman T, Bobbie PO (2005). Classification of arrhythmia using machine learning techniques.

[66] Perai A, Nassiri Moghaddam H, Asadpour S, Bahrampour J, Mansoori G (2010). A comparison of artificial neural networks with other statistical approaches for the prediction of true metabolizable energy of meat and bone meal. Poult Sci.

[67] Singal AG, Mukherjee A, Elmunzer JB, Higgins PDR, Lok AS, Zhu J, Marrero JA, Waljee AK (2013). Machine learning algorithms outperform conventional regression models in predicting development of hepatocellular carcinoma. Am J Gastroenterol.

[68] Friedman JH (2001). Greedy function approximation: A gradient boosting machine. Annal Stat.

[69] Luo W, Nguyen T, Nichols M, Tran T, Rana S, Gupta S, Phung D, Venkatesh S, Allender S (2015). Is demography destiny? Application of machine learning techniques to accurately predict population health outcomes from a minimal demographic dataset. PLoS One.

